# A probability formula derived from serum indicators, age, and comorbidities as an early predictor of dementia in elderly Chinese people

**DOI:** 10.1002/brb3.2236

**Published:** 2021-06-26

**Authors:** Qing Gong, Lianhong Xie, Minghui Bi, Lina Yu

**Affiliations:** ^1^ Department of Geriatrics Shanghai Xuhui Central Hospital Shanghai China

**Keywords:** dementia, elderly screen, homocysteine, low‐density lipoproteins, total cholesterol

## Abstract

**Introduction:**

Blood‐based indicators are potentially economical and a safe method for screening a population for dementia, although their predictive values have not been unequivocally confirmed. The present study proposes a dementia prediction formula based on serum indicators and patient characteristics.

**Methods:**

From January 2016 to December 2018, the data of elderly patients older than 60 years admitted to the Department of Neurology and Geriatrics in our hospital were retrospectively reviewed. A multivariate logistic regression model was applied to verify the patients’ characteristics and serum indicators associated with the risk of dementia. After receiver‐operating characteristic (ROC) curve and area under the ROC curve (AUC) analyses, we propose a dementia prediction formula and cutoff values for the predictive ability of early dementia.

**Results:**

Four thousand seven hundred twenty two elderly patients were enrolled, and the incidence of dementia was 12.0% (565). When patients had ≥8 comorbidities, their risk of developing dementia was 20 times higher than those without comorbidities. After multivariate regression analysis, age (OR: 1.086, *p* < .001) and homocysteine (HCY) concentrations (OR: 1.017, *p* = .003) were proven to be linked to the risk of developing dementia, while total cholesterol (TC) (OR: 0.674, *p* = .005) was a protective factor for dementia. We developed a formula of age + low‐density lipoprotein cholesterol (LDL‐C) + TC + HCY + number of comorbidities as a good predictor of dementia (AUC: 0.79), with a probability (cutoff) value of 0.112 (sensitivity 87.4%, specificity 55.8%, and accuracy 60.5%).

**Conclusions:**

High‐serum HCY and low TC were risk factors for developing dementia. A cutoff value > 0.112 derived from our formula was an excellent predictor for people at a high risk of developing dementia, and may be a potentially useful diagnostic tool for identifying patients at risk for dementia in routine clinical practice.

## Graphical abstract

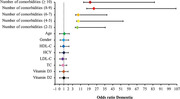

## 1 INTRODUCTION

Dementia is a disease characterized by cognitive decline that affects daily activities and social functioning, and is a great challenge for global health and social care in the 21st century (American Psychiatric Association, 2013). As the world population ages, the incidence of dementia has exponentially increased, particularly in older people. In 2015, it has been estimated that 50 million people had dementia worldwide and by 2050 more than 152 million people are predicted to have this debilitating disease (London WKsC, 2017). In China, the incidence in the population of individuals older than 60 years is 7.2% (global average 6.2%), and the annual incidence rate is 0.625%, accounting for approximately 25% of the global total (Chan et al., 2013; Jin et al., 2015). According to the China cognition and aging study (COAST study), by 2009 there had been 9.2 million people with dementia in China, of which 62.5% were diagnosed with Alzheimer's disease (AD) (Jia et al., 2014). Dementia leads to increased cost for governments, communities, families, and affected individuals, and results in reduced productivity of the economy. It has been estimated that the annual cost of dementia worldwide is about $818 billion (OECD, 2015; WHO, 2017). Existing drugs such as cholinesterase inhibitors and glutamate receptor antagonists can only improve symptoms in the short term but do not delay disease progression (Farlow et al., 2000; Tariot et al., 2004; Winblad et al., 2006). Therefore, early detection, diagnosis, and treatment have become the global consensus of dementia prevention and its treatment.

At the present time, the treatment rate of dementia in China is only 26.9%, with the missed clinical diagnosis rate being as high as 76.8% (e.g., 39% greater than in the Netherlands). Ninety‐three percent of patients with dementia in the community have not been identified (33% higher than in the United Kingdom), and the standardized treatment rate is only 21.3% (less than one third of the United States), which means that the overall level of dementia diagnosis and treatment in China lags well behind high‐income countries (Collerton et al., 2009, p. 1). The Mental State Examination Scale (MMSE) or the Montreal Cognitive Assessment are mainly used to screen for early dementia in China, but scale screening is easily affected by the mental state of the subjects and their surrounding environment, and the assessment accuracy is often poor and follow‐ups are required. A more economical and safe method of population screening would be the collection of accessible tissue samples (such as blood) to screen for predictor indicators.

Studies on the association between some common clinical blood test indicators and dementia have increased in recent years. Measurement of serum lipid profiles is a routine and extensive clinical procedure for the diagnosis and guidance of treatment for patients with dementia. Lipid profiles are considered valuable blood‐based biomarkers because they are readily modifiable factors to potentially slow or prevent the development of dementia. However, published studies on the association between lipid profiles and the risk of developing dementia have to date produced inconsistent results (Beydoun et al., 2011; Raffaitin et al., 2009; Reitz et al., 2010; Solomon et al., 2009). Similarly, as a modifiable indicator, high levels of homocysteine (HCY) have toxic effects on blood vessels and nerves and are associated with the pathogenesis of dementia (Lipton et al., 1997). However, the results of epidemiological prospective cohort studies on serum HCY and dementia risk were inconsistent, with some reporting a positive association (Haan et al., 2007; Seshadri et al., 2002) and others concluding that there was no association (Kim et al., 2008; Luchsinger et al., 2004). In addition, many investigations have suggested a link between vitamin D deficiency and dementia (Annweiler et al., 2011; Landel et al., 2016), and that supplementation with vitamin D derivatives may well reduce the risk of dementia from developing (Dean et al., 2011).

We conducted a large sample real‐world study involving 4722 elderly Chinese patients from January 2016 to December 2018, in order to identify the risk factors for dementia, including demographic characteristics and common serum indicators. We also aimed to develop a dementia prediction formula that could identify elderly patients at high risk of developing dementia.

## 2 MATERIALS AND METHODS

### 2.1 Study population

The study was a retrospective analysis of data acquired from dementia and control elderly patients older than 60 years from January 2016 and December 2018 who were treated in our hospital. Patients with suspected dementia diagnosis were transferred from neurology, geriatrics, or other departments (20%) or admitted for hospitalization and treatment due to acute illness and were subsequently diagnosed with dementia (70%) or have been diagnosed with dementia and the condition aggravated to moderate and severe (10%).

The control patients received therapy for a number of other conditions. Exclusion criteria were individuals with liver failure, a serum creatinine concentration >120 μmol/L, hyperthyroidism or hypothyroidism, disorders of the immune system, or on drugs that alter cholesterol, low‐density lipoprotein cholesterol (LDL‐C), and high‐density lipoprotein cholesterol (HDL‐C) concentrations. The diagnosis of dementia was made according to the 2018 Chinese guidelines for diagnosis and management of dementia and cognitive impairment (I): dementia classification and diagnostic (Zhonghua & Xue, 2018).

The Institutional Review Board approved the employed protocols and waived the requirement for written informed consent.

### 2.2 Data collection

We reviewed the medical histories of patients and documented age, gender, comorbidities, and serum parameters (fasting blood glucose, FBG (mmol/L); HbA1C (mg/dL); total cholesterol, TC (mmol/L); LDL‐C (mmol/L); HDL‐C (mmol/L); HCY (μmol/L); folic acid (mmol/L); vitamin D2 (mmol/L); and vitamin D3 (mmol/L)). Cardiac disease was defined as a history of congestive heart failure; myocardial infarction; angina pectoris or medication with digitalis at any time; hypertension, systolic blood pressure ≥140 mmHg or diastolic blood pressure ≥90 mmHg; use of antihypertensive agents. Diabetes refers to fasting glucose concentration ≥7 mmol/L, postprandial glucose or a 2 h 75 g oral glucose postloaded level ≥1 mmol/L, insulin, or oral hypoglycemic medication. Hypercholesterolemia refers to fasting TC ≥ 6.2 mmol/L or the use of lipid‐lowering drugs (statins, fibrate, bile acid sequestrant). Respiratory disorders refer to asthma, COPD, bronchiectasis, pulmonary fibrosis, or sarcoidosis. Cerebrovascular disease refers to stroke, transient ischemic attacks, aneurysm, or vascular malformations. The history of gastrointestinal disorders refers to the esophagus, stomach, small intestine, large intestine, rectum, pancreas, gallbladder, and liver. Nephrosis was defined as a urine test positive for protein, a blood test positive for protein levels lower than normal, and the clinical detection of edema. A history of fracture was defined as a break in any bone or cartilage. Tumor was defined as having a previous medical history of tumors.

### 2.3 Measurements of serum indicators

Fasting venous blood samples (3 and 4 mL) were collected into tubes containing ethylenediaminetetraacetic acid (EDTA)‐K2 anticoagulant and vacuum‐separated gel blood collection vessels, respectively, and stored at −80°C for subsequent testing. FPG, TC, HDL‐C, LDL‐C, HCY, folic acid, vitamin D2, and vitamin D3 concentrations were measured with an Advia Clinical Chemistry System (Siemens Healthcare, Erlangen, Germany).

### 2.4 Statistical analysis

SPSS version 23 (IBM, USA) was employed to analyze all datasets. Discrete data are given as numbers or percentages and continuous data with a normal distribution as the mean ± SD. To analyze potential risk factors affecting dementia, uni‐ and multivariate logistic regression was employed. Data are given with 95% confidence intervals. The predictive ability of indicators for dementia was evaluated by receiver operating characteristic (ROC) analysis. The cutoff values for indicators were determined by ROC analyses (Youden Index). Variables with statistical significance in the univariate analysis were combined in different ways, to judge the diagnostic effect (ROC) of different combinations. Finally, the optimal combination (the largest area under the ROC curve) was recommended according to the fitting efficiency of different combination models. A statistically significant finding was deemed to be a two‐sided *p‐*value <.05.

## 3 RESULTS

### 3.1 Patient characteristics and baseline information

A total of 4722 elderly patients were included, with an average age of 73.0 ± 15.5 years, and 52.5% were males. Most of the patients were in the Department of Neurology (77.8%). There were 565 patients with dementia, with an incidence rate of 12%. Cerebrovascular disorders, hypertension, and cardiac disorders were the top three comorbidities, accounting for 74.2%, 59.5%, and 38.9%, respectively (Table 1).

**TABLE 1:** General characteristics of the patients

Variables		Patients (*n* = 4722)
Gender	Male	2479 (52.5)
	Female	2243 (47.5)
Age		73.0 ± 15.5
Medical department	Neurology	3672 (77.8)
	Geriatrics	1050 (22.2)
Diagnosis	Dementia	565 (12.0)
	Nondementia	4157 (88.0)
Comorbidities	Cerebrovascular disorders	3502 (74.2)
	Hypertension	2811 (59.5)
	Cardiac disorders	1839 (38.9)
	Diabetes	1192 (25.2)
	Respiratory disorders	1123 (23.8)
	Hyperlipidemia	319 (6.8)
	Tumor	206 (4.4)
	History of fracture	84 (1.8)
	Nephrosis disorders	84 (1.8)
	Gastrointestinal disorders	10 (0.2)
Serum indicators	FBG (mmol/L)	5.9 ± 2.5
	HbA1C (mg/dl)	6.3 ± 1.3
	TC (mmol/L)	4.3 ± 1.1
	HDL‐C (mmol/L)	1.2 ± 0.3
	LDL‐C (mmol/L)	2.3 ± 0.8
	HCY (μmol/L)	17.2 ± 10.5
	Folic acid (mmol/L)	8.5 ± 5.0
	Vitamin D2 (mmol/L)	1.7 ± 3.3
	Vitamin D3 (mmol/L)	14.1 ± 8.0

Abbreviations: FBG, fasting blood glucose; HbA1C, hemoglobin A1C; HCY, homocysteine; HDL‐C, high‐density lipoprotein cholesterol; LDL‐C, low‐density lipoprotein cholesterol; TC, total cholesterol.

### 3.2 Univariate analysis of the general characteristics of dementia

**FIGURE 1:**
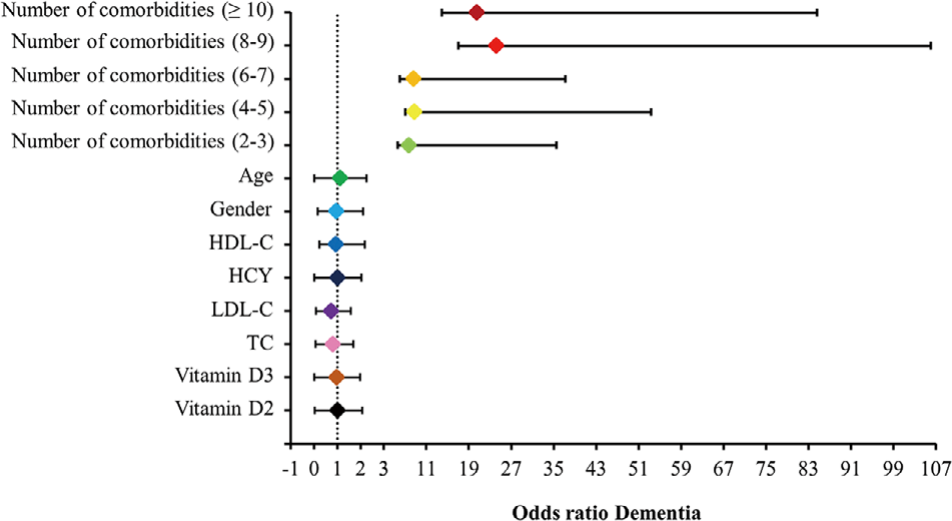
Forest plot of ORs for dementia

Patients with dementia were significantly older than patients without dementia, but there was no difference in gender. Respiratory disorders (OR: 1.411, *p* < .001), fractures (OR: 1.202, *p* < .001), cardiac disorders (OR: 1.123, *p* < .001), hypertension (OR: 1.120, *p* < .001), and cerebrovascular disorders (OR: 1.080, *p* < .001) were associated with a higher risk for the incidence of dementia. However, diabetes did not increase the risk of developing dementia. From the perspective of the number of comorbidities, OR increased with the number of comorbidities. When patients had ≥8 comorbidities, their risk of developing dementia was 20 times higher than those without comorbidities. Even two to three comorbidities increased the odds of dementia by a factor of 7.75. On the other hand, hyperlipidemia was the only indicator we found that was negatively linked to the dementia risk (OR: 0.767, *p* < .001) (Table 2). Figure 1 shows a forest plot of the derived ORs.

**TABLE 2:** Univariate analysis of gender, age, and comorbidities for dementia

		Dementia (*n* = 565)	Nondementia (*n* = 4157)	OR (95% CI)	*p*‐Value
Gender	Male	301 (53.3)	2178 (52.4)	1.0	.694
	Female	264 (46.7)	1979 (47.6)	0.965 (0.809–1.151)	
Age		85.9 ± 8.3	71.2 ± 15.4	1.122 (1.109–1.135)	<.001
Respiratory disorders	No	361 (63.9)	3238 (77.9)	1.0	
	Yes	204 (36.1)	919 (22.1)	1.411 (1.285–1.549)	<.001
Hypertension	No	189 (33.5)	1722 (41.4)	1.0	
	Yes	376 (66.5)	2435 (58.6)	1.120 (1.053–1.192)	<.001
Hyperlipidemia	No	550 (97.3)	3853 (92.7)	1.0	
	Yes	15 (2.7)	304 (7.3)	0.767 (0.672–0.875)	<.001
History of fracture	No	544 (96.3)	4094 (98.5)	1.0	
	Yes	21 (3.7)	63 (1.5)	1.202 (1.087–1.329)	<.001
Nephrosis disorders	No	557 (98.6)	4081 (98.2)	1.0	
	Yes	8 (1.4)	76 (1.8)	0.958 (0.847–1.082)	.488
Cerebrovascular disorders	No	100 (17.7)	1120 (26.9)	1.0	
	Yes	465 (82.3)	3037 (73.1)	1.080 (1.046–1.116)	<.001
Diabetes	No	422 (74.7)	3108 (74.8)	1.0	
	Yes	143 (25.3)	1049 (25.2)	1.0 (0.976–1.026)	.969
Gastrointestinal disorders	No	565 (100)	4147 (99.8)	1.0	
	Yes	0 (0)	10 (0.2)	0.258 (<0.001– >999.9)	.975
Cardiac disorders	No	205 (36.3)	2678 (64.4)	1.0	
	Yes	360 (63.7)	1479 (35.6)	1.123 (1.102–1.143)	<.001
Tumor	No	548 (97)	3968 (95.5)	1.0	
	Yes	17 (3)	189 (4.5)	0.962 (0.919–1.007)	.096
Number of comorbidities	0	3 (0.5)	376 (9.0)	1.0	
	2 ‐ 3	12 (2.1)	194 (4.7)	7.75 (2.16–27.76)	.002
	4 ‐ 5	3 (0.5)	43 (1.0)	8.74 (1.71–44.63)	.009
	6 ‐ 7	27 (4.8)	393 (9.5)	8.60 (2.59–28.58)	<.001
	8 ‐ 9	24 (4.2)	124 (3.0)	24.24 (7.18–81.84)	<.001
	≥ 10	496 (87.7)	3027 (72.8)	20.52 (6.57–64.13)	<.001

Abbreviation: CI, confidence interval.

### 3.3 Univariate analysis of serum indicators for dementia

We also compared serum indicators in patients with and without dementia. The risk of the incidence of dementia was reduced with higher concentrations of TC (OR: 0.804, *p* < .001), LDL‐C (OR: 0.743, *p* < .001), and vitamin D3 (OR: 0.982, *p* = .015), whereas it increased with higher concentrations of HCY (OR: 1.012, *p* = .017) (Table 3).

**TABLE 3:** Analyses of biomarkers for the risk of developing dementia (*n* = 4722)

	Dementia (*n* = 565)	Nondementia (*n* = 4157)	OR (95% CI)	*p*‐Value
Univariate analysis				
FBG (mmol/L)	6.1 ± 2.6	5.9 ± 2.5	1.021 (0.986–1.057)	.244
HbA1C (mg/dL)	6.3 ± 1.2	6.3 ± 1.3	0.953 (0.879–1.032)	.236
TC (mmol/L)	4.1 ± 1.1	4.4 ± 1.1	0.804 (0.734–0.881)	<.001
HDL‐C (mmol/L)	1.2 ± 0.4	1.2 ± 0.3	0.939 (0.712–1.239)	.657
LDL‐C (mmol/L)	2.1 ± 0.7	2.3 ± 0.8	0.743 (0.653–0.844)	<.001
HCY (μmol/L)	18.5 ± 10.3	16.9 ± 10.5	1.012 (1.002–1.021)	.017
Folic acid (mmol/L)	8.3 ± 5.5	8.5 ± 5.0	0.992 (0.971–1.013)	.454
Vitamin D2 (mmol/L)	1.9 ± 3.4	1.6 ± 3.3	1.022 (0.994–1.051)	.131
Vitamin D3 (mmol/L)	13.1 ± 8.3	14.2 ± 7.9	0.982 (0.967–0.996)	.015
Multivariate analysis				
Age			1.086 (1.067–1.105)	<.001
TC			0.674 (0.513–0.885)	.005
HCY			1.017 (1.006–1.028)	.003

Abbreviations: FBG, fasting blood glucose; HbA1C, Hemoglobin A1C; HCY, homocysteine; HDL‐C, High‐density lipoprotein cholesterol; LDL‐C, low‐density lipoprotein cholesterol; TC, total cholesterol.

### 3.4 Multivariate analysis of general characteristics and serum indicators for dementia

Our multivariate regression analysis showed that age (OR: 1.086, *p* < .001) and HCY concentrations (OR: 1.017, *p* = .003) were risk factors for developing dementia, while TC (OR: 0.674, *p* = .005) was a protective factor against developing this condition (Table 3).

### 3.5 The predictive ability of LDL‐C, TC, and HCY concentrations, and their combinations with age and the number of comorbidities in predicting dementia

**FIGURE 2:**
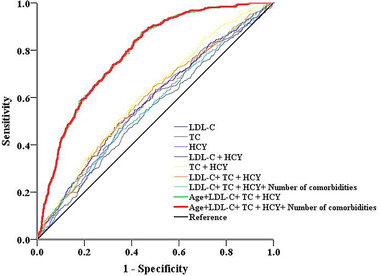
ROC curves of the predictive models of dementia in elderly patients Abbreviations: HCY, homocysteine; HDL‐C, high‐density lipoprotein cholesterol; LDL‐C, low‐density lipoprotein cholesterol; TC, total cholesterol.

We performed ROC analysis of a large group of patients (*n* = 4722) and found that age + LDL‐C + TC + HCY + number of comorbidities was a good predictor of dementia (AUC: 0.79), with a cutoff value of 0.112 (sensitivity 87.4%, specificity 55.8%, accuracy 60.5%) (Table 4, Figure 2).

**TABLE 4:** The cutoff value, sensitivity, specificity, and accuracy of serum indicators, and their combination with patient characteristics to predict dementia in ROC analysis

	Cutoff value	Sensitivity (%)	Specificity (%)	Accuracy (%)	ROC
LDL‐C	2.18	58.1 (53.7–62.5)	53.0 (51.4–54.6)	53.6 (52.1–55.1)	0.54 (0.51–0.58)
TC	4.15	56.4 (51.9–60.7)	55.4 (53.8–57.1)	55.6 (54.0–57.1)	0.57 (0.53–0.60)
HCY	14.5	61.0 (55.4–66.4)	52.5 (50.2–54.8)	53.7 (51.6–55.8)	0.57 (0.54–0.60)
LDL‐C + HCY	0.154	44.5 (38.9–50.1)	66.4 (64.2–68.6)	63.2 (61.1–65.2)	0.56 (0.53–0.60)
TC + HCY	0.156	49.2 (43.6–54.9)	65.5 (63.2–67.6)	63.1 (61.0–65.1)	0.58 (0.55–0.62)
LDL‐C + TC + HCY	0.144	64.0 (58.5–69.3)	51.0 (48.7–53.4)	53.0 (50.8–55.1)	0.59 (0.56–0.62)
LDL‐C + TC + HCY+ number of comorbidities	0.155	59.9 (54.5–65.3)	57.1 (54.8–59.3)	57.5 (55.4–59.6)	0.61 (0.58–0.64)
Age + LDL‐C + TC + HCY	0.126	83.9 (79.9–88.0)	58.7 (56.4–60.9)	62.4 (60.3–64.4)	0.79 (0.76–0.81)
Age + LDL‐C + TC + HCY + number of comorbidities	0.112	87.4 (83.7–91.0)	55.8 (53.6–58.1)	60.5 (58.4–62.5)	0.79 (0.76–0.81)

Abbreviations: AUC, area under the curve; HCY, homocysteine; HDL‐C, high‐density lipoprotein cholesterol; LDL‐C, low‐density lipoprotein cholesterol; TC, total cholesterol.

We developed a formula (*p* = exp (−10.2858 + 0.1074 × age + 0.3922 × LDL‐C − 0.3901 × TC + 0.0113 × HCY + 0.0785 × number of comorbidities)/(1 + exp (−10.2858 + 0.1074 × age + 0.3922 × LDL‐C − 0.3901 × TC + 0.0113 × HCY + 0.0785 × number of comorbidities)) to be used to identify people who were at an increased risk of developing dementia. These results indicated that a combination of age + LDL‐C + TC + HCY + number of comorbidities may be a potential candidate formula to predict dementia.

## 4 DISCUSSION

In the present study, high HCY concentrations and low TC levels were closely related with the risk of developing dementia among Chinese elderly people. In view of the need for blood‐based screening to identify people most at risk of developing this condition, our study has proposed a formula (including age, LDL‐C, TC, HCY, and number of comorbidities) as a predictive tool to screen out patients at a higher risk of developing dementia at the community level, thus providing the basis for further accurate diagnosis.

Table 2 shows that 25% of patients with dementia had DM, which was not significantly higher than in the group of patients without dementia. A previous study suggested that DM was linked to less severe forms of cognitive dysfunctions, which can occur in young adults, adolescents, and older patients, but further cognitive decline over time was regarded as generally slow over the course of many years (Biessels & Despa, 2018). However, diabetes‐related decrements of cognitive dysfunctions have been confined to neurodegenerative changes associated with aging (Biessels et al., 2008), which might explain that age but not DM appeared as a significant risk factor for dementia in our analyses.

As a result of the analysis of the general characteristics of patients, we found that age was a risk factor that was uncontrollable. Age was clearly the biggest risk factor for developing dementia, and most patients with sporadic dementia start to get ill after the age of 65. Epidemiological studies (Chan et al., 2013) in different countries worldwide have confirmed that the incidence and prevalence of dementia increases with age. The results of a meta‐analysis revealed that the incidence of dementia doubled every 10 years after the age of 60 (Prince et al., 2013). It is worth noting that dementia is not an inevitable result of aging, and aging itself is not the only reason for the development of dementia.

Vascular risk factors are considered to be important indicators of dementia prevention (de Bruijn et al., 2015). Since lipid components represent potential prevention targets that are relatively easy to modify, it is of great clinical importance to explore their relationships with the risk of developing dementia. To date, studies on any link between dyslipidemia and dementia have produced inconsistent results. The age at which a patient's blood lipid levels are measured, and the length of follow‐up may explain these differences. High cholesterol levels were shown to increase the risk of dementia, primarily in studies that measured lipid levels in middle age and/or followed the subjects over time until late in their lives. In contrast, short‐term follow‐up blood lipid measurement studies of patients in old age or those who did not reach this age with the highest prevalence of dementia, either found no association (Beydoun et al., 2011; Li et al., 2005) or sometimes an inverse relationship with the risk of dementia (Hayden et al., 2006; Mielke et al., 2005). Our study found that TC was a protective factor for dementia in a large sample of elderly people, and that low TC levels increased the risk of developing dementia. Cholesterol is one of the most important components of neurons and is essential for the development and maintenance of neuronal plasticity and functions (Pfrieger, 2003). Low cholesterol concentrations may be a symptom of dementia progression (Panza et al., 2009) and may herald the onset of dementia (van den Kommer et al., 2009). Even a drop in the cholesterol concentration, 9 years before dementia has developed, can affect the diagnosis (Mielke et al., 2005). TC levels may be reduced over time, but the rate of decline was much greater in patients who eventually experienced impairment of cognition (Stewart et al., 2007). In addition, a high TC concentration was associated with a lower mortality of older people (Brescianini et al., 2003), and it can thus be speculated that raised cholesterol concentrations give rise to better health than for people who have low cholesterol levels. In particular, these people may have better liver functions because a low TC concentration may reflect liver disease (Brescianini et al., 2003). Several studies in Chinese populations also support this view (Lv et al., 2016; Zhou et al., 2018).

Previously published literature has reported that high HCY levels are independent risk factors for cognitive dysfunction, cerebrovascular disease, and atherosclerosis (Tay et al., 2006). High levels of HCY have been linked with an elevated risk of individuals developing cardiovascular disease and all‐cause deaths (Bates et al., 2010), but the relationship between HCY and dementia or cognitive deterioration has not been consistently demonstrated (Ho et al., 2011). Our study found that a high HCY concentration is a risk factor for dementia, which is consistent with the results of previous domestic and foreign studies (Van Dam & Van Gool, 2009 ). An increased HCY concentration may be associated with cognitive decline and the mechanisms involved may be related to direct neurotoxic or cerebrovascular damage. An increased concentration of HCY induces a cascade stress response, leading to intracranial arteriolosclerosis, which eventually induces an insufficient cerebral blood supply that leads to atrophy of the brain. High HCY concentrations can improve the sensitivity of neurons to excitatory poisons, promote apoptosis of neurons, and affect nerve conduction (Samoylenko et al., 2010). Interestingly, a recent cross‐sectional study (Cheng et al., 2014) found that both low and high cholesterol concentrations might be harmful to cognitive health in people with normal HCY levels. However, in people with high HCY concentrations, HCY has an overwhelming effect on cognition, regardless of the cholesterol concentration. This finding suggests that cholesterol and HCY may interact in the cognitive functions of an aged population. Both cholesterol and HCY concentrations can effectively be controlled by existing drugs. In 2012, the US Food and Drug Administration (FDA) added possible cognitive adverse reactions (including memory problems) to statin prescription information (FDA, 2012). In terms of the risk of dementia, the cholesterol‐lowering drugs commonly used in the elderly should be taken with caution. However, since serum HCY reflects the functional status of the B‐group vitamins, folic acid, vitamin B12 and B6, the risk of developing dementia by this factor is modifiable by supplementing B vitamins in the diet (Smith et al., 2018).

Dementia is a global epidemic and early detection of patients at risk of dementia has become an internationally recognized priority. Blood‐based predictive indicators are attractive options in the clinic because they are safe, reliable, simple to use, and cost‐effective for screening. For the screening of AD, a number of blood‐based biomarkers have initially demonstrated the efficacy of distinguishing AD from matched controls in the elderly. Neocortical Aβ (extracellular β‐amyloid) burden (NAB) is a good predictor of the progress of AD. One study recommended predicted human NAB level measurements based on the molecular characteristics of blood (sensitivity: 79.6%; specificity: 82.4%; AUC: 87.6%) (Burnham et al., 2014). In addition, it was also found that the success rate of MMSE and 25(OH)D3 combination in predicting mild cognitive impairment (MCI) and AD reached 98% (Ouma et al., 2018), suggesting that this combination can support the clinical diagnosis of MCI and the mild, medium, and serious stages of AD. Our study proposes a formula based on blood test indicators to predict dementia (sensitivity 87.4%; specificity 55.8%; AUC 79%). This formula is simple and easy to use. The blood test indicators (TC, LDL‐C, and HCY) contained in the formula are low‐cost routine tests. The prediction formula can be used as a screening tool for a broad population at the community level to facilitate the identification of patients who could potentially benefit from further more invasive or more expensive confirmatory tests for diagnosis (such as cerebrospinal fluid analysis or positron emission tomography (PET)).

There are a number of limitations to our research that should be considered. First, the patients in our study were all Han people who live in Shanghai. Although this study analyzed a large cohort of patients, caution is needed when extending our conclusions to people of other races and cities. Second, we made no comparisons between the different clinical types and different levels of cognitive impairment of dementia. Third, there may be a reverse causal relationship between lipid levels and dementia, and patients with dementia may be more likely to suffer from eating disorders and malnutrition, which may lead to lower cholesterol levels in the body. Unfortunately, the design of a cross‐sectional study makes it impossible to explore causality. Further prospective studies are needed to provide unequivocal evidence of causality.

In conclusion, this real‐world cross‐sectional study of a large sample size found that high HCY concentrations and low TC concentrations were independent risk factors for dementia in elderly patients. The formula of age + LDLC + TC + HCY + number of comorbidities predicted dementia and may serve as a cost‐effective tool for its early detection in people at a risk of developing dementia, and who could benefit from further invasive or indeed expensive confirmatory tests.

## CONFLICT OF INTEREST

The authors declare that there is no conflict of interest.

## AUTHOR CONTRIBUTIONS

*Study concept and design*: All authors. *Acquisition of data*: Qing Gong, Minghui Bi, and Lina Yu. *Analysis and interpretation of data*: All authors. *Drafting of the manuscript*: Qing Gong, Minghui Bi, and Lina Yu. *Critical revision of the manuscript for important intellectual content*: Lianhong Xie.

## Data Availability

The data that support the findings of this study are available from the corresponding author upon reasonable request.
